# Alpha-Adrenergic Agonists Stimulate Fluid Secretion in Lacrimal Gland Ducts

**DOI:** 10.1167/iovs.61.14.3

**Published:** 2020-12-01

**Authors:** Dóra Szarka, Gréta Elekes, Orsolya Berczeli, Eszter Vizvári, László Szalay, Chuanqing Ding, László Tálosi, Edit Tóth-Molnár

**Affiliations:** 1 Department of Ophthalmology, University of Szeged, Szeged, Hungary; 2 Pharmacology & Pharmaceutical Sciences, Ophthalmology, University of Southern California, Los Angeles, California, United States; 3 Department of Pharmacognosy, University of Szeged, Szeged, Hungary

**Keywords:** lacrimal gland (LG), duct cell, adrenergic regulation, α-adrenergic

## Abstract

**Purpose:**

The role of adrenergic innervation in the regulation of lacrimal gland (LG) ductal fluid secretion is unknown. The Aim of the present study was to investigate the effect of adrenergic stimulation on fluid secretion in isolated LG duct segments and to study the underlying intracellular mechanisms.

**Methods:**

Fluid secretion of isolated mouse LG ducts was measured using video-microscopy. Effect of various adrenergic agonists (norepinephrine, phenylephrine, and isoproterenol) on fluid secretion as well as inhibitory effects of specific antagonists on adrenergic agonist-stimulated secretory response were analyzed. Changes in intracellular Ca^2+^ level [Ca^2+^_i_] were investigated with microfluorometry.

**Results:**

Both norepinephrine and phenylephrine initiated a rapid and robust fluid secretory response, whereas isoproterenol did not cause any secretion. Phenylephrine-induced secretion was completely blocked by α_1D_-adrenergic receptor blocker BMY-7378. The endothelial nitric oxide synthase (eNOS) inhibitor L-NAME or guanylyl cyclase inhibitor ODQ reduced but not completely abolished the phenylephrine-induced fluid secretion, whereas co-administration of Ca^2+^-chelator BAPTA-AM resulted in a complete blockade. Phenylephrine stimulation induced a small, but statistically significant elevation in [$Ca_{i}^{2+}$].

**Conclusions:**

Our results prove the direct role of α_1_-adrenergic stimulation on LG ductal fluid secretion. Lack of isoproterenol-induced fluid secretory response suggests the absence of β-receptor mediated pathway in mouse LG ducts. Complete blockade of phenylephrine-induced fluid secretion by BMY-7378 and predominant inhibition of the secretory response either by L-NAME or ODQ suggest that α-adrenergic agonists use the NO/cGMP pathway through α_1D_ receptor. Ca^2+^ signaling independent from NO/cGMP pathway may also play an at least partial role in α-adrenergic induced ductal fluid secretion.

Tear film is a substantial protector of the ocular surface. A predominant amount of the aqueous layer is produced by the lacrimal gland (LG).^1^ Similar to other exocrine glands, LG consists of acini and ducts.^2^ Most of the research activities were focusing on the function of acinar cells and much less efforts have been paid to the investigation of the ductal system, even though an important role of the duct cells in LG function has been assumed for a long time.^3^^,^^4^ Lack of experimental methods suitable to examine the function of LG ducts hindered the availability of studies focusing solely on the role of the duct system. An isolated duct model was developed in our laboratory with the modification of the method used in pancreas duct research.^5^^,^^6^ Using this model and a video-microscopy technique, experimental evidence of fluid secretion of rabbit LG ducts was given, confirming the important role of ducts in tear secretion.^7^ The isolated duct model is also suitable for the investigation of the regulatory mechanisms of the duct system.^5^^,^^7^^–^^9^

Autonomic regulation of the ductal function is not fully explored. Parasympathetic pathways are the main regulatory system of the LG, whereas sympathetic effects have been supposed to play indirect role through blood flow regulation.^10^^–^^12^ There is increasing evidence, however, that sympathetic stimulation - apart from the hemodynamic effects - plays a direct and important role in the protein secretion of the LG.^13^^,^^14^ Although earlier reports suggested that both α_1_ and β_1_-adrenergic agonists could result in protein secretory response in whole LG pieces of mouse and rat, the role of α_1_-adrenergic receptors is expected to be more relevant.^15^^,^^16^ Furthermore, intracellular mechanisms mediating α-adrenergic stimulation in LGs involve additional pathways beside the conventional route through activation of phospholipase C.^17^ This conception is supported by the well documented fact that the dominant α-adrenergic receptor subtype presents in the LG is the α_1D_ and not the most common α_1A_ or α_1B_ subtypes.^18^^,^^19^ Intracellular mechanisms of α_1D_-adrenergic receptor activation are not clearly understood. Additionally, involvement of the NO/cGMP pathway was suggested in the phenylephrine-induced protein secretion of rat LG.^18^ All these results were obtained from studies investigating the effect of adrenergic stimulation on acinar cells or on whole LG pieces. However, the effect of adrenergic stimulation as well as the intracellular mechanisms underlying this process in ducts of LGs are completely unknown.

Therefore, the aim of the present study was to investigate the effect of adrenergic stimulation on fluid secretion of isolated LG duct segments and to study the intracellular mechanisms underlying adrenergic stimulation.

Parts of the results in this paper have been presented in abstracts in the Annual Meetings of the Association for Research in Vision and Ophthalmology (Berczeli O., et al. IOVS 2017; 58(8): 2256; Tóth-Molnár E., et al. IOVS 2018; 59(9): 4923).

## Materials and Methods

### Animals

Mouse exorbital LGs dissected from 12 to 16 week old wild type FVB/N mice (a total of 56 animals) were used throughout the study. Animals were narcotized intraperitoneally with ketamine (80 mg/kg) and xylazine (10 mg/kg), and euthanized with pentobarbital overdose (100 mg/kg).

All experiments were conducted in compliance with the ARVO Statement for the Use of Animals in Ophthalmic and Vision Research. The protocol was approved by the Ethical Committee for the Protection of Animals in Research of the University of Szeged, Szeged, Hungary, and conformed to the Directive 2010/63/EU of the European Parliament.

### Solutions and Chemicals

Media and its supplements for LG duct isolation and culture (Dulbecco's modified Eagle medium, McCoy's 5A tissue culture medium, fetal calf serum, glutamine, and bovine serum albumin), phenylephrine, isoprenaline, propranolol, phentolamine, norepinephrine, carbachol (carbamylcholine chloride), endothelial nitric oxide synthase (eNOS) inhibitor L-NAME, guanylyl cyclase inhibitor ODQ, and α_1D_-adrenergic receptor inhibitor BMY-7378 were purchased from Sigma-Aldrich Corp. (Budapest, Hungary). Collagenase was purchased from Worthington Biochemical Corp. (Lakewood, NJ, USA). FURA2-AM was purchased from Invitrogen (Waltham, MA, USA). The compositions of solutions used in our experiments are summarized in the Table. The standard HCO_3_^−^/CO_2_^−^ buffered solution was gassed with 95% O_2_/5% CO_2_ at 37°C.

**Table.** Composition of Solution

	Content of Solutions				
Compound		HCO_3_^−^ / CO_2_^−^ Buffered Solution	Isolation Solution	Storage Solution	Culture Solution
NaCl, mM		115			
KCl, mM		5			
MgCl_2_, mM		1			
CaCl_2_, mM		1			
D-Glucose, mM		10			
NaHCO_3_, mM		25			
Dulbecco's Modified Eagle Medium			X	X	
Collagenase, U/mL			100		
Bovine serum albumin, mg/mL			1	0.03	
McCoy's 5A Tissue Culture Medium					X
Fetal calf serum, vol/vol %					10
Glutamine, mM					2

### Isolation of Ducts From Mouse LGs

Mouse LG interlobular ducts were isolated as previously described by our laboratory.^5^ Briefly, LGs were dissected and transferred to a sterile small flat-bottom glass flask containing cold (4°C) storage solution. Isolation solution was injected into the interstitium of the glands and the tissue pieces were transferred to a glass flask containing 2 mL of isolation solution. Following a 15 minute incubation period in a shaking water bath at 37°C, isolation solution was removed and 5 mL of fresh cold storage (4°C) solution was added to the flask. LG tissue samples were transferred to a glass microscope slide and viewed under stereo-microscope. Following the microdissection of the ducts, the isolated duct segments were transferred to the culture solution in a Petri dish. Isolated ducts were cultured overnight in a 37°C incubator gassed with 5% CO_2_.

### Measurement of Ductal Fluid Secretion

Video-microscopic method was used for the measurement of ductal fluid secretion. The technique was originally described for the investigation of pancreatic ducts and was adapted by our laboratory for the measurement of ductal fluid secretion.^6^^,^^7^ In brief, ends of the isolated ducts seal after 8 to 10 hours of incubation. Secretory processes of the epithelial cells result in luminal volume (LV) increase of the ducts as the closed luminal space fills with the secreted fluid. The change in ductal volume can be analyzed with video-microscopy. Scion Image (Scion Corporation, Frederick, MD, USA) software was used to analyze and calculate changes in the LV.

### Measurement of Intracellular Ca^2+^

Ca^2+^-sensitive fluorescent dye FURA 2AM (5 µM) was used for the measurement of intracellular Ca^2+^ concentration [Ca^2+^]_i_ as described earlier.^5^ Changes in [Ca^2+^]_i_ were measured using an imaging system (Xcellence; Olympus, Budapest, Hungary). Four to 5 small areas (region of interests [ROIs]) of 5 to 10 cells in each intact duct were excited with light at 340 nm and 380 nm, and the 380 / 340 fluorescence emission ratio were measured at 510 nm. One [Ca^2+^]_i_ measurement was obtained per second.

### Statistical Analysis

For the analysis of ductal fluid secretion, effects of the stimulatory agents (phenylephrine, isoproterenol, and norepinephrine) were considered as “fixed effects.” The effect of the individual “duct” and the “duct and effects of phenylephrine/isoproterenol/ norepinephrine interaction” (we presumed the individual duct-dependent effects of the stimulatory compounds) were taken into account as random effects. For the investigation of the inhibitory effect of L-NAME, ODQ, and BMY-7378, data were expressed as the percent change of the LV above baseline LV (baseline LV was considered 1.0). A mixed ANOVA model was used for statistics, by using SigmaPlot version 12.5 (Systat Software Inc., San Jose, CA, USA), results were presented as means ± SEM. A *P* value of < 0.05 was regarded as significant.

## Results

### Effect of Adrenergic Agonists on Fluid Secretion of LG Ducts

**Figure 1.**
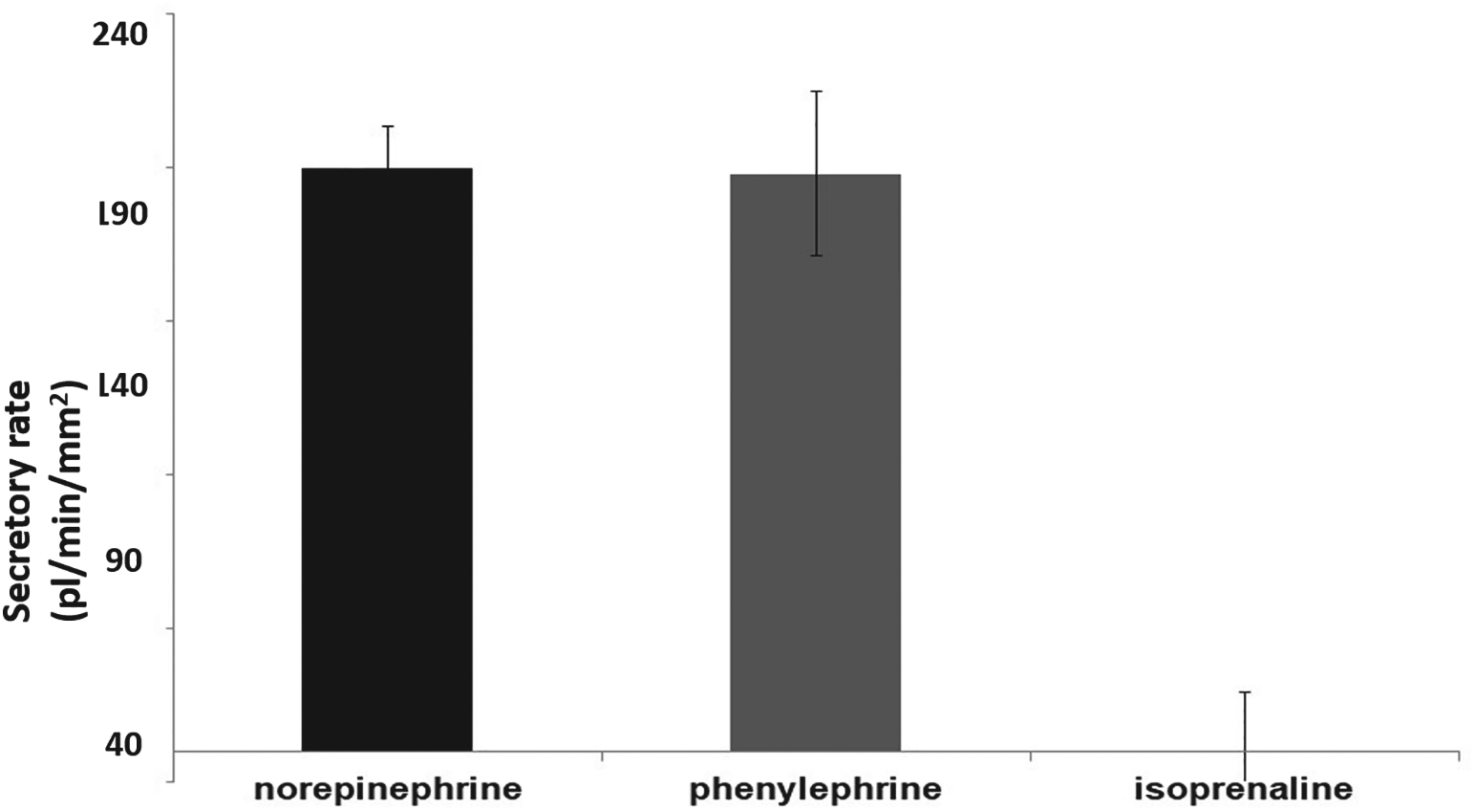
Effect of different adrenergic agonists on ductal fluid secretion in isolated lacrimal gland ducts. Isolated lacrimal gland ducts were stimulated with norepinephrine (10 µM), or with phenylephrine (10 µM) in the presence of propranolol (1 µM) or with isoproterenol (200 µM) in the presence of phentolamine (10 µM). Secretory response of ducts was measured with video-microscopy. Changes in relative luminal volume (Vr) are shown. Data were obtained at least from six ducts isolated from three different animals in each series and are presented as means ± SEM.

Isolated mouse LG ducts were used for the investigation of the effect of various adrenergic agonist on ductal fluid secretion. In the first series of experiments, ducts were stimulated with various concentrations (5, 10, or 20 µM) of the natural adrenergic agonist norepinephrine (noradrenaline) to determine the secretory response and dose-response relationship. Norepinephrine stimulates both α- and β-adrenergic receptors causing a complete adrenergic upset. Application of norepinephrine initiated a dose-dependent, rapid fluid secretory response (5 µM: 120.7 ± 19.1 pl/min/mm^2^; 10 µM: 189.6 ± 13.9 pl/min/mm^2^; and 20 µM: 181.5 ± 11.7 pl/min/mm^2^ in the first 10 minutes of stimulation). The most effective concentration of norepinephrine proved to be 10 µM (Fig. 1), higher concentration (20 µM) did not result in further increase in the secretory response of the investigated ducts. To analyze the role of various adrenergic receptors in the observed secretory response, effects of selective α_1_ and β_1_-adrenergic stimulations were investigated. In the α_1_-adrenergic studies, ducts were stimulated with phenylephrine. Various concentrations (5, 10, or 20 µM) were used to determine the secretory response and dose-response relationship. To ensure the blockade of β-adrenergic receptors, phenylephrine was administered in the presence of β-adrenergic antagonist propranolol (1 µM). Phenylephrine stimulation caused a rapid fluid secretory response in the isolated duct segments (Fig. 1). Supplementary Video S1 demonstrates the effect of phenylephrine stimulation on ductal fluid secretion. The most effective concentration of phenylephrine found to be 10 µM (secretory rates in the first 10 minutes of stimulation: 5 µM: 116.5 ± 19.1 pl/min/mm^2^; 10 µM:187.8 ± 26.8 pl/min/mm^2^; and 20 µM: 182.1 ± 22.5 pl/min/mm^2^). Therefore, concentration of 10 µM was used throughout the additional phenylephrine experiments. It is important to mention that no statistically significant difference was detected between the extent of the fluid secretory rates evoked by phenylephrine in the presence of propranolol versus norepinephrine (*P* = 0.42) and the kinetics of these stimulated secretions were also similar.

Effect of β-adrenergic stimulation on ductal fluid secretion was also investigated. β-adrenergic agonist isoproterenol was administered in the presence of α-adrenergic antagonist phentolamine (10 µM) to ensure the selective β-adrenergic stimulation. Isoproterenol failed to elicit any detectable secretory effect in all applied concentrations (secretory rates in the first 10 minutes of stimulation: 50 µM: −0.2 ± 11.4 pl/min/mm^2^; 100 µM: 0.1 ± 17.1 pl/min/mm^2^; and 200 µM: −0.8 ± 19.7 pl/min/mm^2^). Figure 1 exhibits secretory result of the highest isoproterenol concentration applied (200 µM).

### Effect of α_1D_-Adrenergic Receptor Antagonist BMY-7378 on Phenylephrine-Evoked Ductal Fluid Secretion

**Figure 2.**
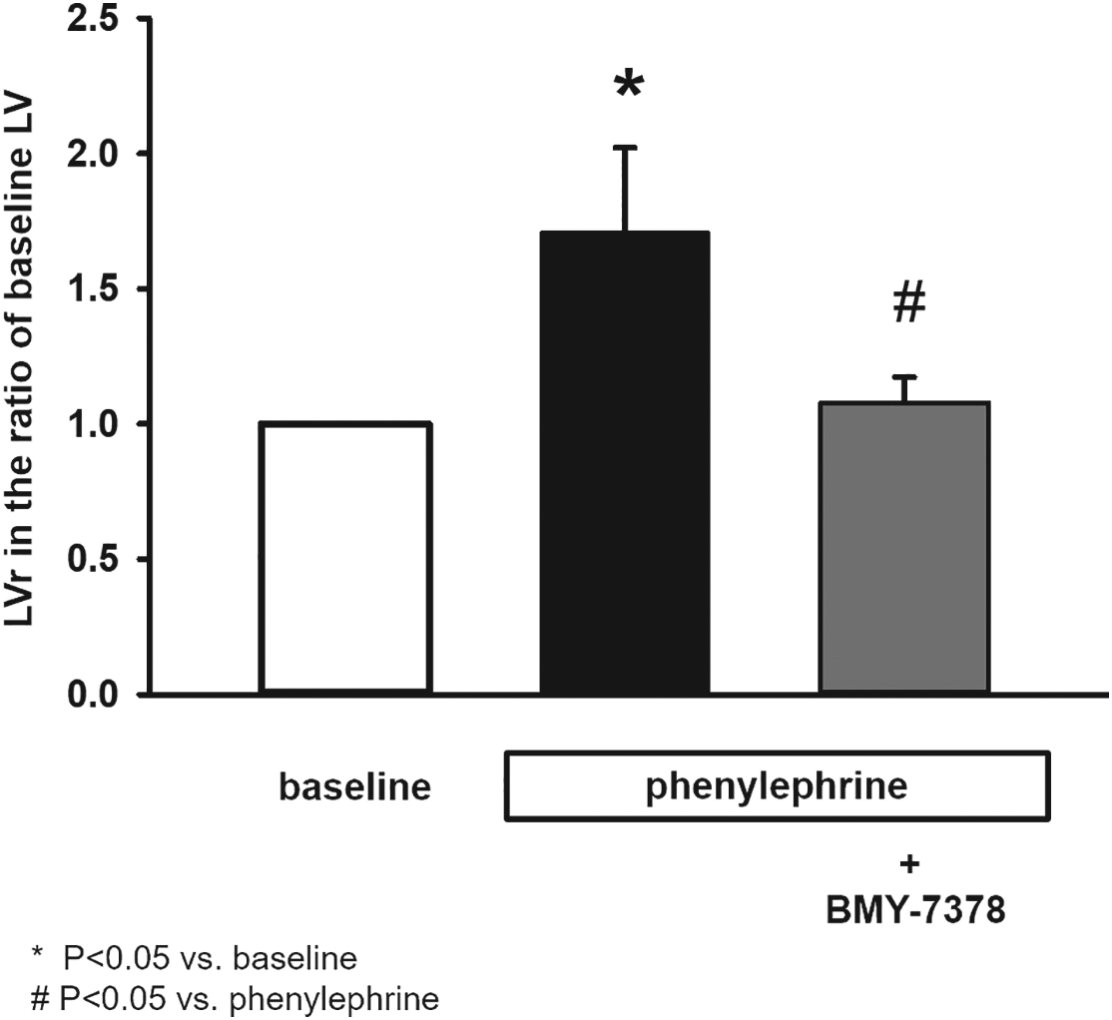
Effect of BMY-7378 pretreatment on phenylephrine induced secretory response of isolated lacrimal gland ducts. Isolated ducts were stimulated with phenylephrine (10 µM) either in the presence or in the absence of α_1D_-receptor antagonist BMY-7378 (100 µM). Secretory response of ducts was measured with video-microscopy. Changes in relative luminal volume (LVr) are shown. Data were obtained at least from six ducts isolated from three different animals in each series and are presented as means ± SEM.

Secretory response of isolated ducts suggested to be clearly due to the stimulation of α-adrenergic receptors in our experiments. Earlier studies demonstrated that α-adrenergic receptor subtype present in the acinar epithelial cells of LG is the α1D.^18,19^ Therefore, we investigated the effect of α1D-adrenergic blockade to explore the subtype of the involved receptors in the isolated mouse LG ducts. Duct segments were pre-incubated with different doses of selective α1D receptor antagonist BMY-7378 (1, 10, 100, or 200 µM) for 30 minutes and then phenylephrine (10 µM) was added to the superfusate. BMY-7378 reduced phenylephrine-induced ductal fluid secretion in a dose-dependent manner (1 µM: 58.27 ± 7.12% above baseline LV; 10 µM: 42.24 ± 6.51% above baseline LV; 100 µM: 7.64 ± 9.68% above baseline LV; and 200 µM: 7.69 ± 8.71% above baseline LV; maximal inhibition at 100 µM [baseline LV means unstimulated state]). The difference between baseline LV and the LV measured following phenylephrine stimulation in the presence of 100 µM BMY-7378 was statistically not significant (*P* = 0.081). Therefore, administration of 100 µM BMY-7378 completely abolished phenylephrine-induced ductal fluid secretion proving the role of α_1D_-adrenergic receptors in the observed secretory response (Fig. 2).

### Effect of eNOS Inhibitor L-NAME and Guanylyl Cyclase Inhibitor ODQ on Phenylephrine-Induced Ductal Fluid Secretion

**Figure 3.**
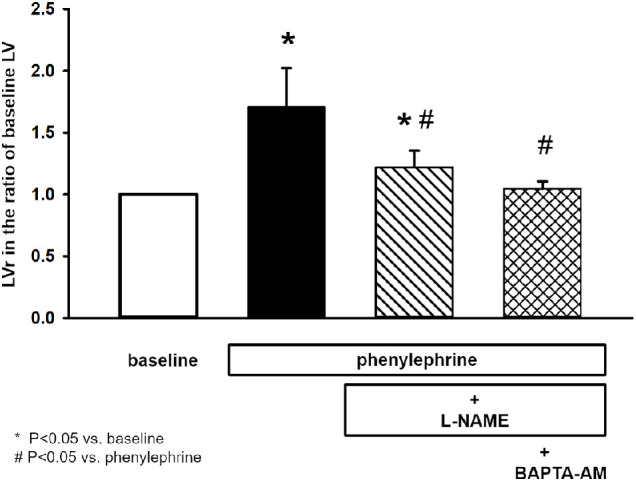
Effect of L-NAME and L-NAME/BAPTA-AM pretreatment on phenylephrine induced secretory response of isolated lacrimal gland ducts. Isolated ducts were stimulated with phenylephrine (10 µM) either in the absence of eNOS inhibitor L-NAME or in the presence of L-NAME (100 µM) alone or combined with Ca^2+^ chelator BAPTA-AM (10 µM). Secretory response of ducts was measured with video-microscopy. Changes in relative luminal volume (LVr) are shown. Data were obtained at least from six ducts isolated from three different animals in each series and are presented as means ± SEM.

Because the mechanisms underlying α_1D_-adrenergic receptor stimulation involve the NO/cGMP pathway, the role of this intracellular pathway was investigated in the next series of experiments. LG ducts were pre-incubated with different doses of eNOS inhibitor L-NAME (1, 10, 100, or 200 µM) for 30 minutes and then 10 µM of phenylephrine was added to the bath. Phenylephrine-evoked ductal fluid secretion was reduced by L-NAME in a dose-dependent manner (1 µM: 53.01 ± 8.2% above baseline LV; 10 µM: 33.5 ± 10.02% above baseline LV; 100 µM: 21.82 ± 13.52% above baseline LV; and 200 µM: 22.14 ± 14.10% above baseline LV; maximal inhibition at 100 µM). However, even at the maximal inhibition effect of L-NAME, a significant difference (*P* = 0.023) was found between baseline LV and LV measured following phenylephrine stimulation in the presence of L-NAME (Fig. 3). These results suggest that administration of L-NAME reduced, but not completely abolished the phenylephrine-induced fluid secretion of isolated LG ducts.

**Figure 4.**
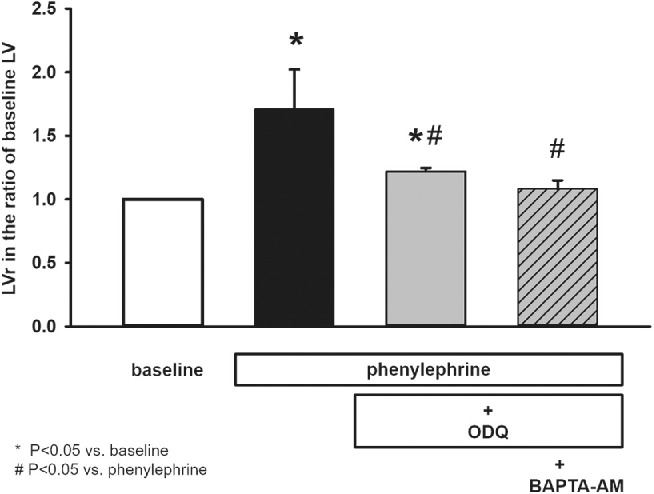
Effect of ODQ and ODQ/BAPTA-AM pretreatment on phenylephrine induced secretory response of isolated lacrimal gland ducts. Isolated ducts were stimulated with phenylephrine (10 µM) either in the absence of guanylyl cyclase inhibitor ODQ or in the presence of ODQ (10 µM) alone or combined with Ca^2+^ chelator BAPTA-AM (10 µM). Secretory response of ducts was measured with video-microscopy. Changes in relative luminal volume (LVr) are shown. Data were obtained at least from six ducts isolated from three different animals in each series and are presented as means ± SEM.

In the next series of experiments, LG ducts were pre-incubated with different doses of guanylyl cyclase inhibitor ODQ (0.1, 1, 10, or 100 µM) for 30 minutes before administration of phenylephrine (10 µM). Inhibition of guanylyl cyclase with ODQ decreased phenylephrine-induced LV increase in a dose dependent manner (0.1 µM: 70.90 ± 9.07% above baseline LV; 1 µM: 55.28 ± 10.01% above baseline LV; 10 µM: 21.78 ± 2.97% above baseline LV; and 100 µM: 23.12 ± 5.20% above baseline LV). Maximal inhibition occurred at 10 µM ODQ concentration. Although the inhibitory effect of ODQ was visible, a significant difference (*P* = 0.0008) was proved between baseline LV and LV measured following phenylephrine stimulation in the presence of ODQ (Fig. 4). Effect of ODQ administration was similar to that L-NAME produced in the previous experiments: it reduced, but not completely inhibited phenylephrine-induced ductal fluid secretion.

### Phenylephrine-Evoked Ca^2+^ Signaling in Isolated LG Duct Segments

Although α_1D_ receptor blockage with BMY-7378 completely abolished phenylephrine-induced ductal fluid secretion, inhibition of eNOS or guanylyl cyclase considerably reduced but could not block it completely. We hypothesized in the background of this phenomenon that the elevation of [Ca^2+^]_i_ as a consequence of α_1D_-adrenergic receptor activation may contribute to the fluid secretion of the ducts.

**Figure 5.**
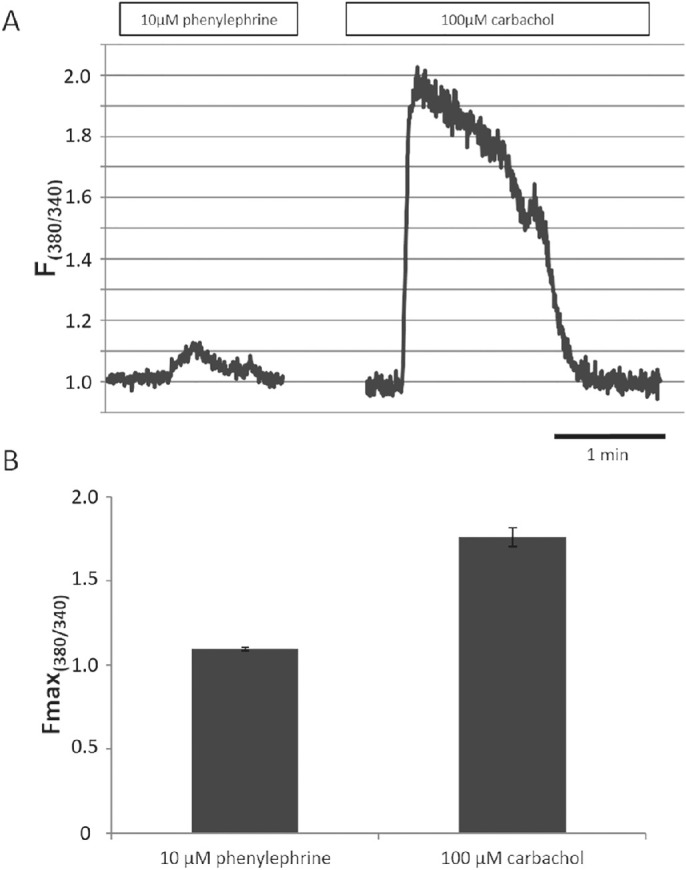
Effects of phenylephrine and carbachol on [Ca^2+^]_i_ in cells of isolated mouse lacrimal gland ducts. Ducts were preincubated with Ca^2+^-sensitive fluorescent dye FURA 2AM (5 µM) and then stimulated either with phenylephrine (10 µM) or with carbachol (100 µM). (**A**) Representative recordings of the micro-fluorescence experiments: effect of phenylephrine (10 µM) on [Ca^2+^]_i_ in lacrimal gland duct cells (*left curve*). Carbachol (100 µM) was used as a positive control in these experiments (*right curve*) (F_380/340_: 380/340 nm fluorescence emission ratio). (**B**) Maximum values of the 380/340 nm fluorescence emission ratios (F_max(380/340)_: maximum value of the 380/340 nm fluorescence emission ratio).

To investigate this theory, in the next series of experiments, [Ca^2+^]_i_ change was measured in response to phenylephrine stimulation. In these experiments, applied concentration of phenylephrine was 10 µM similarly to the fluid secretion experiments. Stimulation of α-adrenergic receptors by phenylephrine resulted in a small, but statistically significant increase in [Ca^2+^]_i_ (*P* = 0.012). The extent of this increase was much smaller (Fig. 5), compared to the response we observed previously during carbachol stimulation in epithelial cells of isolated mouse LG ducts.^9^

### Effect of Ca^2+^ Chelator BAPTA-AM on Phenylephrine-Induced Ductal Fluid Secretion

Phenylephrine-induced ductal fluid secretion was measured in BAPTA-AM pretreated ducts in order to investigate the role of Ca^2+^ in the secretory process. Phenylephrine stimulation resulted in 169.21 ± 22.5 pl/min/mm^2^ fluid secretory rate in duct cells preloaded with 10 µM of BAPTA-AM. Although this value was slightly lower compared to the secretory rate evoked by phenylephrine alone (187.8 ± 26.8 pl/min/mm^2^), no statistically significant difference could be demonstrated in the fluid secretion between BAPTA-AM-treated and non-treated ducts (*P* = 0.052).

### Effect of Co-Administration of L-NAME or ODQ With Ca^2+^ Chelator BAPTA-AM on Phenylephrine-Induced Ductal Fluid Secretion

In contrast to BMY-7378, eNOS inhibitor L-NAME considerably reduced but not completely abolished phenylephrine-induced ductal fluid secretion. To investigate the potential role of phenylephrine-evoked elevation of [Ca^2+^]_i_, the effect of L-NAME on phenylephrine-induced secretion was investigated in the presence of intracellular Ca^2+^-chelator BAPTA-AM. In these experiments, isolated ducts were pre-incubated with the most effective dose of L-NAME (100 µM) and BAPTA-AM (10 µM). Co-administration of L-NAME and BAPTA-AM completely blocked phenylephrine-induced ductal fluid secretion (LV change: 2.1 ± 4.8% above baseline LV, *P* = 0.67).

Based on similar considerations (i.e. further investigation of reduced but not completely abolished phenylephrine-induced ductal fluid secretion in ODQ experiments) phenylephrine-induced secretion was also studied in the combined presence of ODQ and intracellular Ca^2+^-chelator BAPTA-AM. Isolated ducts were pre-incubated with ODQ (10 µM) and BAPTA-AM (10 µM) in these experiments. A complete inhibition of phenylephrine-induced ductal fluid secretion was observed following co-administration of ODQ and BAPTA-AM: change of LV was negligible and nonsignificant compared to baseline value (LV change: 3.1 ± 2.5% above baseline LV, *P* = 0.63).

## Discussion

Tear secretion is regulated by the autonomic nervous system. Besides the generally accepted decisive role of parasympathetic innervation, there is accumulating experimental evidence about the direct effect of sympathetic regulation of LG function.^13^^–^^20^ The presumptive role of adrenergic regulation was confirmed in studies focusing on protein secretion of acinar cells and whole LG pieces from rat and mouse.^15^^,^^16^^,^^20^^,^^21^ In the present study, role of adrenergic effect in the regulation of LG ductal fluid secretion is demonstrated. Application of the natural adrenergic transmitter norepinephrine (or noradrenaline) induced a rapid and robust fluid secretion in the isolated ducts. Considering the intense response observed, sympathetic stimulation may have more functional significance than previously believed. As norepinephrine stimulates both α and β-adrenergic receptors, the pharmacological background of the observed secretory response was investigated. Stimulation of α-adrenergic receptors with phenylephrine in the presence of β-adrenergic blocker propranolol resulted in a pronounced ductal fluid secretion similar to that observed during application of norepinephrine. In contrast, no detectable fluid secretion was observed by the activation of β-adrenergic receptors with isoproterenol in the presence of α-adrenergic antagonist phentolamine. These results are in accordance with a previously published study, where high density of α-adrenergic receptors and very weak presence of β-adrenergic receptors were found in LG ducts by immunostaining.^20^ Our results strongly suggest the involvement of the sympathetic nervous system in the regulation of ductal fluid secretion. Decisive role of α-adrenergic stimulation in the sympathetic neurotransmission was demonstrated because no β-adrenergic induced fluid secretion could be observed. The α-adrenergic receptor subtype present in the acinar epithelial cells of LG is the α_1D_, not the more common α_1A_ or α_1B_. Selective α_1D_ receptor blocker BMY-7378 could completely abolish phenylephrine-induced ductal fluid secretion in our experiments, proving the involvement of the same receptor subtype in the sympathetic innervation of LG ducts. The intracellular mechanisms underlying α_1D_-adrenergic receptor stimulation was found to be more complex and less clearly clarified compared to α_1A_ and α_1B_ subtypes.^22^ To elucidate the intracellular mechanisms underlying α-adrenergic stimulated ductal fluid secretion, the role of NO/cGMP pathway was investigated. Both eNOS inhibitor L-NAME and guanylyl cyclase inhibitor ODQ reduced but not entirely blocked phenylephrine-evoked ductal fluid secretion. These findings differed from the results obtained by Hodges et al. in rat LG acinar cells where application of either L-NAME or ODQ resulted in a complete blockade of phenylephrine-induced protein secretion.^18^ An additional and obviously NO/cGMP pathway-independent mechanism was supposed in the background of the observed partial blockade. Because α-adrenergic stimulation is generally linked to Ca^2+^ signaling, the effect of phenylephrine on [Ca^2+^]_i_ and ductal fluid secretion was investigated.^23^ Although phenylephrine stimulation resulted in a small but statistically significant elevation of [Ca^2+^]_i_, no statistically significant difference could be demonstrated in the fluid secretion between the Ca^2+^-chelator BAPTA-AM-treated and nontreated ducts.

To specify the role of the observed increase in [Ca^2+^]_i_ in the α-adrenergic stimulation-enhanced fluid secretion, further series of experiments were performed. In these experiments, Ca^2+^ signaling was excluded by co-administration of intracellular Ca^2+^-chelator BAPTA-AM either with L-NAME or ODQ. Under these circumstances, complete blockade of phenylephrine-induced ductal fluid secretion could be reached demonstrating the apparent role of NO/cGMP pathway-independent Ca^2+^ signaling mechanism.

Although the main intracellular event in the fluid secretion evoked by phenylephrine is the activation of the guanylyl-cyclase-cGMP pathway even though minor elevation of [Ca^2+^]_i_ plays some role. Blockage of the cGMP pathway alone markedly reduced but not completely abolished fluid secretion, whereas in combination with depletion of [Ca^2+^]_i_ resulted in complete stoppage. On the other hand, because the elevation of [Ca^2+^]_i_ was small, distraction of Ca^2+^ itself did not result in significant reduction in fluid secretion, although some tendency of lower secretory rates could be noticed (without reaching the statistically significant level).

In conclusion, our data strongly suggest the direct role of α-adrenergic stimulation in LG ductal fluid secretion. Lack of isoproterenol-induced fluid secretory response and the similar secretory effects of norepinephrine and phenylephrine suggest that the determining adrenergic pathway is via α_1D_-adrenergic receptors in mouse LG ducts. Inhibition of phenylephrine-induced ductal fluid secretion by α_1D_-adrenergic receptor antagonist or by reduction of fluid secretion by either eNOS or guanylyl cyclase inhibitors suggest that α-adrenergic agonists use the NO/cGMP pathway through α_1D_ receptor stimulation to increase fluid secretion, but involvement of a NO/cGMP pathway-independent Ca^2+^ signaling mechanism is also assumed.

## Supplementary Material

Supplement 1
